# Supplementary material for: Tailor-made biochar systems: Interdisciplinary evaluations of ecosystem services and farmer livelihoods in tropical agro-ecosystems
Source: PLoS One. 2022 Jan 28;17(1):e0263302. doi: 10.1371/journal.pone.0263302 (PMC8797206; doi:10.1371/journal.pone.0263302)
Supplement: S1 File — (DOCX) [file pone.0263302.s001.docx]

**Supplementary information for: Tailor-made biochar systems: Interdisciplinary evaluations of ecosystem services and farmer livelihoods in tropical agro-ecosystems**

Severin-Luca Bellè^1^, Jean Riotte^2,3^, Norman Backhaus^1,4^, Muddu Sekhar^3,5^, Pascal Jouquet^3,6^ and Samuel Abiven^7,8*^

^1^ Department of Geography, University of Zurich, Zurich, Switzerland

^2^ Géosciences Environnement Toulouse, Université Paul-Sabatier, IRD, CNRS, Toulouse, France

^3^ Indo-French Cell for Water Science, Indian Institute of Science, Bangalore, Karnataka, India

^4^ University Research Priority Programme (URPP) Global Change and Biodiversity, University of Zurich, Zurich, Switzerland

^5^ Department of Civil Engineering, Indian Institute of Science, Bangalore, Karnataka, India

^6^ Institut d’écologie et des sciences de l’environnement, IESS-Paris UMR Sorbonne Université, UPEC, CNRS, IRD, INRAe, FEST Team, Bondy, France

^7^ Laboratoire de Géologie, Département de Géosciences, CNRS – École normale supérieure, PSL University, Institut Pierre Simon Laplace, Paris, France

^8^ CEREEP-Ecotron Ile De France, ENS, CNRS, PSL University, St-Pierre-lès-Nemours, France

* Corresponding Author:

Email: abiven@biotite.ens.fr (SA)

**Supplementary information for the in-depth, qualitative expert and farmer interviews conducted during fieldwork in Berambadi watershed, southwestern Karnataka, India in 2016**

Topic guide used for conducting expert interviews with scientists, NGOs, private companies and local experts during fieldwork in India.

**Topic guide for expert interviews**

**1. Introduction**

Meet up with interviewee, get seated and create a relaxed atmosphere through initial exchange. Thank the interviewee for his/her interest in the project and for taking time for the interview.

**2. Presentation of project topic**

Before starting the interview, a short introduction of the interviewer, and of the topic and the aim of the study will be given. Thereafter, the interviewee is asked to pose any question regarding the purpose or procedure of the interview or the research(er).

**3. Agreement**

Emphasize that the information gathered in the interview will be treated objectively and anonymous, so that no information is retraceable to any interviewee.

Subsequently, ask for permission of recording the interview with a voice recorder or digital device (and if necessary explain the intention of recording).

**4. Interviewee's profile**

Gender

Age

Education

Relation to agriculture

Motivation for participation

**5. Interview specification**

Interview Nr.

Interviewer

Date

Duration

Interview situation (interaction, relationship, difficult situations, etc.)

**6. Topic guide**

| **Main question(s)** | **Check-list for additional/detailed information -** only ask if not mentioned  **Memos -** questions can be phrased according to situation | **Follow-up and Probes** |
| --- | --- | --- |

| **Part I - Agriculture in Karnataka** | | |
| --- | --- | --- |
| What is the importance of agriculture in Karnataka (and India)? | - Economic perspective (workforce, rural employment, etc.) - Social perspective (for society or farmers) - **Current Problems and Solutions** | - Is there anything to add from a ... perspective? - Could you please specify this? - What do you mean by that? - Is there anything more to tell about...? - What is your opinion on this topic? - What would be the best solution for this...? |
| How agricultural production does looks like in the state of Karnataka? | - Agricultural inputs (fertiliser, machinery, small-scale applications) - Geographical distribution (agro-ecological zones, climate) |  |
| **Part II - Farming / Farmers (focus on farming practices in general)** | | |
| How does farming look like? What do farmers do in general? | - Traditional, knowledge-based and small-scale farming practises - Practises for soil cultivation / land management and soil fertilisation | - Can you tell me a bit more about ... practises? - How did it come to it? |

| **Part III - Agricultural residue management** | | | | |
| --- | --- | --- | --- | --- |
| How do farmers use plant material or animal excreta (residues) on their farms? | - Usage versus non-usage (reasons for not using it) - Traditional versus new technologies - Compost (input material, method of composting, suitability for local context) - Vermicomposting (awareness of method, production) - Biochar applications (existence, pros & cons, material) | | - According to your expertise, what is the best management practise? - Why do you favour a certain management practise (reasons)? - Can you tell me more about this method? - Can you explain that to me in more detail? - How did it come to it? | |
| What do farmers think about these organic materials / residues? | - Various point of views (waste, resource) - Usage (heating, animal fodder, for fields) - Importance for their livelihood | |  |  |
| What are suitable farming practices when it comes to agricultural residue management? | - Under various environmental and socio-economic contexts (e.g. knowledge, soil fertility) - Sustainability of management practises (application rate, impact, duration of effects) - Openness to new technologies (expectations, fears, education) | |  |  |
| **Part IV - Environmental context of agriculture** | | | | |
| What impact does the local climate have upon agriculture in the different agro-ecological zones? | - Adaption of agricultural practises - Suitable farming practises to manage organic material | | - What method is most useful according to your opinion / expertise? - Why? | |
| What impact do various levels of soil fertility have upon agriculture? | - Adaption of agricultural practises of soil cultivation/amendment - Agricultural residue management practise for soil amendment | |  |  |
| **Part V - Conclusion** | | | | |
| Is there anything important you would like to add? | | - Check list | | - Anything else to add? - Did you miss key dimensions? |

**7. Note**

This final topic guide slightly deviates from the first draft due to changes during data collection. The major changes were done after the pre-test, minor changes or rephrasing of questions after individual interviews if necessary. The content addressed by question however was kept.

Topic guide used for conducting in-depth, qualitative interviews with farmers (n = 29) of Berambadi watershed, southwestern Karnataka, India during fieldwork.

**Topic guide for farmer interviews**

**1. Introduction**

Meet up with interviewee, get seated and create a relaxed atmosphere through initial exchange. Get introduced through the translator. Thank the interviewee for his/her interest in the project and for taking time for the interview.

**2. Presentation of project topic**

Before starting the interview, a short introduction of the interviewer, and of the topic and the aim of the study will be given. Thereafter, the interviewee is asked to pose any question regarding the purpose or procedure of the interview or the research(er).

**3. Agreement**

Emphasize that the information gathered in the interview will be treated objectively and anonymous, so that no information is retraceable to any interviewee.

Subsequently, ask for permission of recording the interview with a voice recorder or digital device (and if necessary explain the intention of recording).

**4. Interviewee's profile**

Gender

Age

Profession and education

Family status

Information on farm (size, topography)

Information on agricultural production (soil, crop, irrigation)

Motivation for participation

**5. Interview specification**

Interview Nr.

Interviewer

Date

Duration

Location of interview (house, field, village, etc.)

Interview situation (interaction, relationship, difficult situations, etc.)

**6. Topic guide**

| Main question(s) **/ Stimulus** | **Check-list for additional/detailed information -** only ask if not mentioned  **Memos -** questions can be phrased according to situation | **Specializing questions / explanations** | **Follow-up and Probes** |
| --- | --- | --- | --- |
| **Concluding question:** Is there anything important you want to add to the ... part on ... ? | | | |

| **Part I - Importance of agriculture** | | | |
| --- | --- | --- | --- |
| What are you thinking about practicing farming? | - For yourself (identification), your family or community - For the environment/nature, for tradition and culture - Future of farm | - What is most important for you about farming? - Do you want to change anything on your farm / your farming practises? | - Why is this so? - Can you tell me more about that? - And... ? - How did it came to this? |
| Is there anything important you want to add to the first part on your vision of agriculture? | | | |

| **Part II - Farming (focus on agricultural production and practices)** | | | |
| --- | --- | --- | --- |
| Crop  Can you explain me how you grow the crop XY from the preparation of the land until the harvest?  Practices | - Reason for choice (climate, soil, tradition and knowledge) - Inputs in farming (fertiliser, pesticide, machinery) - Productivity per area, yields of crops | - Why do you cultivate this crop on your land? - Are you using many external inputs? - Would you use organic material instead of external inputs? - Could you imagine using organic farming practises to increase yields? | - Anything else you cultivate? - Can you tell me your decision behind it? - How did it come to this? - Why is this so? Why not? - If so, can you explain it to me in more detail? |
|  | - Socio-cultural perspective (tradition, knowledge and education) - Socio-economic perspective (value, feasibility) - Environmental perspective (soil quality, crop yields) - Harvest (method, loss of organic material) | - Where did you learn this practise? - Why do you use this practise (value)? - What effects do the farming practises have upon your fields and soils? - What on crop productivity? - Do you lose a lot of material during harvest? | - Can you explain me how these practises work in more detail? - How did it come to this? - Can you give me examples of effects? - Can you explain me this in more detail? - If so, can you explain me why? |
| Is there anything important you want to add to the second part on your farming / agricultural practices? | | | |

| **Part III - Agricultural residues as a resource for sustainable agriculture (focus on usage/management practices of agricultural residues on-farm)** | | | |
| --- | --- | --- | --- |
| How do you use plant material and animal excreta (residues)? | - Usage versus non-usage - Farming practises (leaving it on field, animal fodder, composting, burning) - Alternative usage (fuel, construction material, etc.) - Material as a resource | - If you do not use the material, what are you doing with it? - What for? - Do you think organic material is a resource for you? | - Can you give me an example? - Can you explain why you are not using it? - Can you give specific examples of management practises? - How did it come to this? |
| Do you use the organic material (plants, excreta) for soils?  *(specifically looking at applications to soils and for fertilization of crops)* | - Material (input material, mixture) - Method of preparing organic material (crop residue burning, composting, vermicomposting, etc.) - Reason for practises | - What material do you use for soils? - How do you apply it to soils? - What is the reason behind putting XY to the soil? | - How did it come to this? - Can you explain it to me in more detail? |
| Do you use cow dung manure or composting?  *(as a traditional way of using organic materials on-farm)* | - Material (plants, animal excreta (urine, dung)) - Practises (mixing, decomposition process, application) - Knowledge and education - Experience - Benefits and constraints | - How do you prepare your compost? - Can you show me? - From where did you learn about composting? - Have you done it for long? - Do you see changes after you have applied compost to your fields (soil, plant)? | - Can you explain me in detail how you do it? - What do you do next? - Can you give me an example? |
| Is there anything important you want to add to the third part on practices of agricultural residue management and application to soils? | | | |

| **Part IV - New farming technologies (focus on openness towards vermicomposting and biochar applications)** | | | |
| --- | --- | --- | --- |
| Introduction:  *Start with asking if they have ever heard of vermicomposting respectively biochar as an agricultural practice.*   - *If so: Ask for more detail on the story (practises, context) before continuing with the questions below* - *If not: Continue with the introduction of the essentials of vermicomposting and biochar (use the laminated pictures to illustrate the technologies)*    - *Subsequently start with the questions below after the introduction* | | | |
| What do you think about vermicomposting?  Would you use it on your farm? | General benefits and considerations   - Socio-cultural perspective (knowledge transfer, prestige) - Socio-economic perspective (crop productivity, returns, expected losses) - Environmental perspective (soil health, waste management)   *(At this point: Name some benefits like education, increase in soil fertility and yield, knowledge transfer, easy application)*   - Openness toward technologies (hopes and fears) | - What benefits do you wish to have from such practises? When would you apply it? - What are your considerations / doubts? | - Can you give me an example? - Can you specify this? - How did it come to that? |
| What do you think about biochar?  Would you use it on your farm? | General benefits and considerations   - Socio-cultural perspective (knowledge transfer, prestige) - Socio-economic perspective (crop productivity, returns, expected losses) - Environmental perspective (soil health, waste management)   *(At this point: Name some benefits like education, increase in soil fertility and yield, knowledge transfer, easy application)*   - Openness towards technologies (hopes and fears) | - What benefits do you wish to have from such practises? - When would you apply it? - What are your considerations / doubts? | - Can you give me an example? - Can you specify this? |
| Is there anything important you want to add to the fourth part on the expectations of the introduction of new agricultural technologies? | | | |
| **Part V - Conclusion** | | | |
| - Is there anything you want to add that has not been discussed yet? Any topics you want to raise you think are important? | | | |
